# Formation of the hollow nanopillar arrays through the laser-induced transformation of TiO_2_ nanotubes

**DOI:** 10.1038/s41598-020-77309-2

**Published:** 2020-11-19

**Authors:** Jakub Wawrzyniak, Jakub Karczewski, Piotr Kupracz, Katarzyna Grochowska, Emerson Coy, Adam Mazikowski, Jacek Ryl, Katarzyna Siuzdak

**Affiliations:** 1 Centre of Plasma and Laser Engineering, Institute of Fluid-Flow Machinery, Polish Academy of Sciences, Fiszera 14 st., 80-231 Gdańsk, Poland; 2 Department of Solid-State Physics, Gdańsk University of Technology, Gabriela Narutowicza 11/12 st., 80-233 Gdańsk, Poland; 3 NanoBioMedical Centre, Adam Mickiewicz University, Wszechnicy Piastowkiej 3 st., 61-614 Poznań, Poland; 4 Department of Metrology and Optoelectronics, Gdańsk University of Technology, Gabriela Narutowicza 11/12 st., 80-233 Gdańsk, Poland; 5 Department of Electrochemistry, Corrosion and Materials Engineering, Gdańsk University of Technology, Gabriela Narutowicza 11/12 st., 80-233 Gdańsk, Poland

**Keywords:** Characterization and analytical techniques, Design, synthesis and processing, Microscopy, Optical spectroscopy, Surface patterning, Nanofabrication and nanopatterning, Nanostructures, Structural properties, Synthesis and processing

## Abstract

In the following article, we present a simple, two-step method of creating spaced, hollow nanopillars, from the titania nanotube arrays via pulsed laser-treatment. Due to the high ordering of the structure, the prepared material exhibits photonic properties, which has been shown to increase the overall photoefficiency. The optical and morphological changes in the titania nanotubes after pulsed laser-treatment with 532, 355, and 266 nm wavelengths in the 10–50 mJ/cm^2^ fluence range are studied. The investigation reveals, that by using appropriate wavelength and energy, the number of surface defects, geometrical features, or both can be tailored.

## Introduction

Due to rising worldwide energy demand and increasing awareness about the dangers of using fossil fuels, more and more attention is being paid to renewable sources of power. The titanium dioxide nanotubes (TiO_2_NTs) have been excessively studied for years since their first synthesis by Assefpour-Dezfuly et al.^1^ due to their excellent photocorrosion resistance^2^, non-toxicity, and outstanding optical properties^3^. Moreover, the TiO_2_NTs exhibit very high surface area and have easily tuneable nanotube length^4^, spacing^5,6^, and pore size^7–9^, all influencing properties of the nanotube array. Because of that, they have found immense success in research involving gas sensors^10^, filtering systems^11^, solar cells^12^, and photocatalysis^13,14^.

However, one of the biggest drawbacks of TiO_2_NTs is their wide bandgap of 3.2 eV limiting their absorption to the UV range, which represents only a small part of the solar spectrum. To successfully employ titania nanotubes in solar-driven applications, various methods of widening absorption band have been discussed in the literature. Modifications with non-metals or organics primarily focus on employing narrow bandgap materials which help in exciton generation over a broader light spectrum^15,16^. On the other hand, doping with noble metals, which strongly interact with light in the visible spectrum through plasmonic resonance, allows the injection of hot-electrons into nanotube arrays improving their overall photoactivity^17,18^.

Interestingly though, structural and morphological changes have also been shown to be a key factor in bandgap engineering^19^. The synthesis of TiO_2_NTs with the high surface-to-volume ratio results in improved radiation harvesting. Simultaneously, optimization of the length of the TiO_2_NTs is crucial to avoid excessive recombination of generated charge carriers^20^.

It should be highlighted that those methods do not rely on the incorporation of foreign atoms into the structure of titania, but rather modify existing one to broaden the absorbance spectra. It is done through so-called self-doping and relies heavily on the creation of structural defects such as oxygen vacancies and employs various methods, e.g. microwave or laser irradiation^21–24^. Likewise, the additional absorbance bands can be created or widened by adjusting TiO_2_NTs spatial distribution, similarly to how it is done in photonic structures^5^.

Recently, the use of titania nanotubes as photonic crystal (PC) arrays gains increased traction in literature^18,25–27^. Due to the high refractive index of TiO_2_ structures, and regular spatial distribution of the nanotubes, the TiO_2_NTs are clear candidates for use as photonic materials. Coupled with the scattering of incident light on top of the tubular layer, the total material absorption could be greatly improved while retaining their charge percolation capabilities^28–30^.

In our work, we present a blueprint for the creation of an array of empty, evenly spaced, self-standing TiO_2_ nanopillars. The proposed smart laser treatment of an open tube TiO_2_NTs results in their closing and formation of the tight cap over the hollow tube. Although they exhibit photonic properties by themselves, they can also serve as a base for further modifications, allowing encapsulation of different materials inside, potentially serving as a shell in novel core–shell type of photonic nanostructures.

## Results and discussion

### Morphology

**Figure 1:**
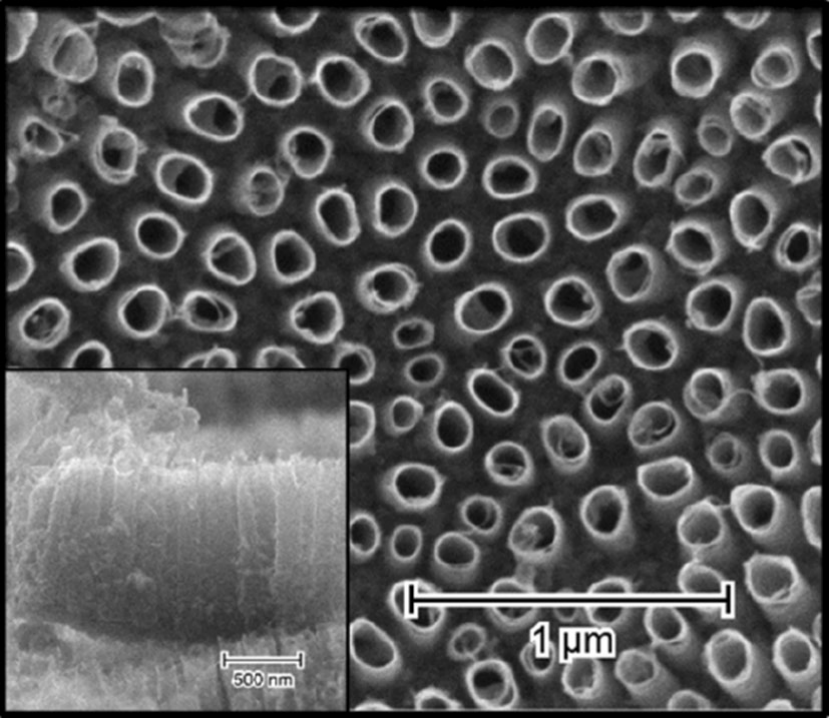
SEM image of the anodized TiO_2_NTs with their cross-section shown in the inset.

**Figure 2:**
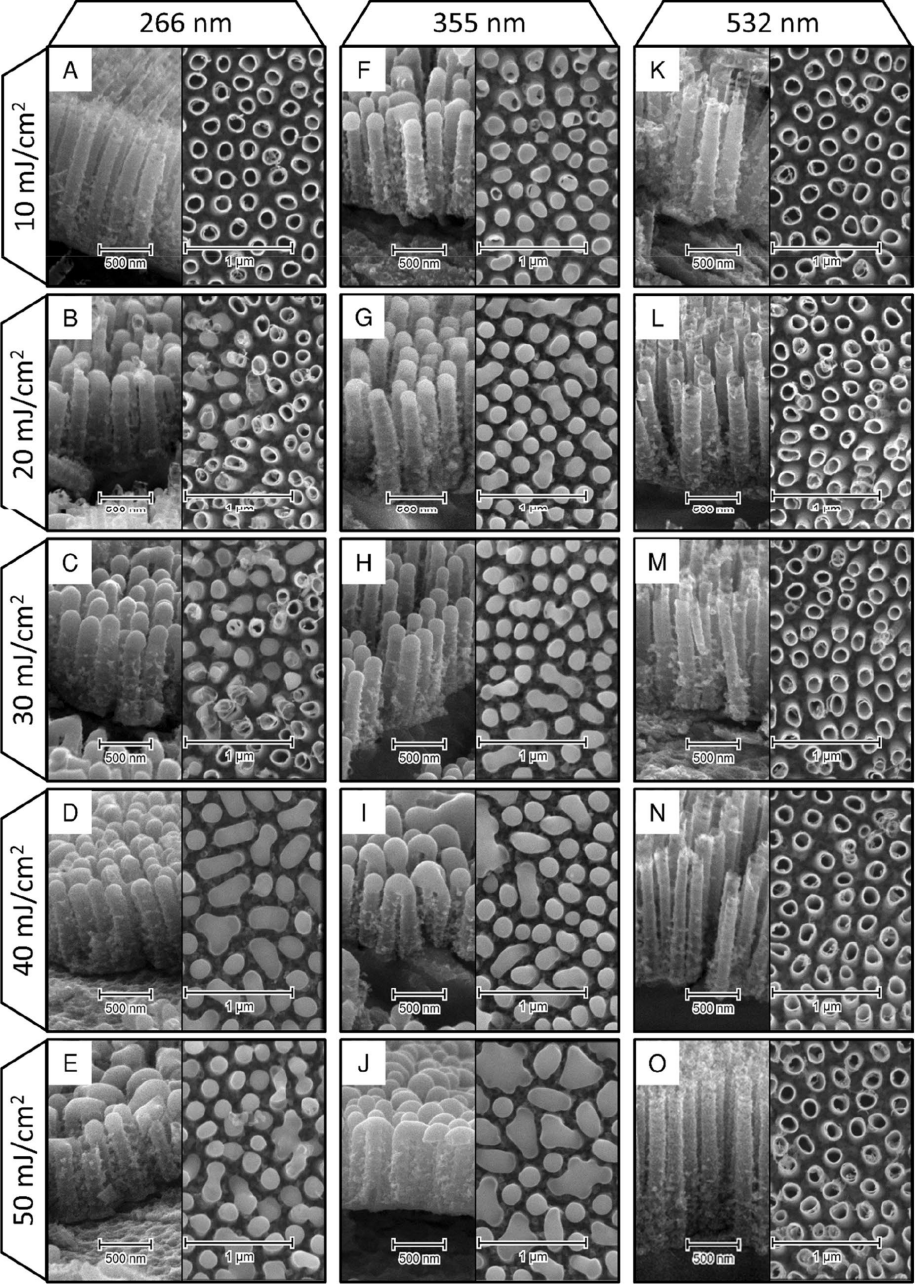
SEM images of the laser-modified TiO_2_NTs. The columns show different wavelengths used (266, 355, and 532 respectively) while rows represent fluence used (10–50 mJ/cm^2^).

As-anodized TiO_2_NTs have an inner radius of about 60 nm, the wall thickness of 12 nm, they are evenly distributed with the average distance between them of 70 nm, and have 1 μm in length (Fig. 1). The calcination process does not affect their morphology in any way, and they retain all their geometrical features. The effects of laser irradiation of different wavelengths within a fluence range of 10–50 mJ/cm^2^ are presented in Fig. 2. The first column shows the effects of modification with 4th harmonics generator crystal (λ = 266 nm), representing photon energy (~ 4.6 eV) well above the bandgap of the material (E_bg_ = 3.3 eV). In the case of the lowest fluence used, no meaningful changes to TiO_2_NTs morphology can be observed (Fig. 2A). However, as the pulse energy rises, some nanotube tops begin to melt, forming connected platforms made of two or three closest nanotubes, resulting in non-uniform surface morphologies. Although the degree of their melting in the upper region varies clearly, the height of the TiO_2_NTs modified with λ = 266 nm changes only slightly with the energy used. While TiO_2_NTs irradiated with 20 mJ/cm^2^ are about 750 nm in length, those treated with 50 mJ/cm^2^ are only about 150 nm shorter.

Considering laser-modifications with 3rd harmonics and photon energy (3.5 eV) close to the bandgap of the material, the degree of changes seems to be much more uniform across energies used (Fig. 2F–J). In the case of the lowest fluence applied, the nanotubes tops melt slightly inwards, forming half-open cylinders. Regarding fluences of 20 and 30 mJ/cm^2^, the upper parts of the nanotubes are fully closed and the vast majority of the nanotubes are self-standing, not merging with neighboring ones. Using higher energies results in gradually more interconnections between TiO_2_NTs. The nanotubes have shortened to about 840 nm after irradiation with 10 mJ/cm^2^, and were approximately 65 nm shorter each time the fluence was increased, down to 575 nm at 50 mJ/cm^2^.

Modification with photon energies (2.3 eV) much lower than the electron bandgap did not result in any changes in overall nanotubes length within the investigated range of fluences. There were, however, visible distortions in the nanotube openings when fluence 50 mJ/cm^2^ was reached.

**Figure 3:**
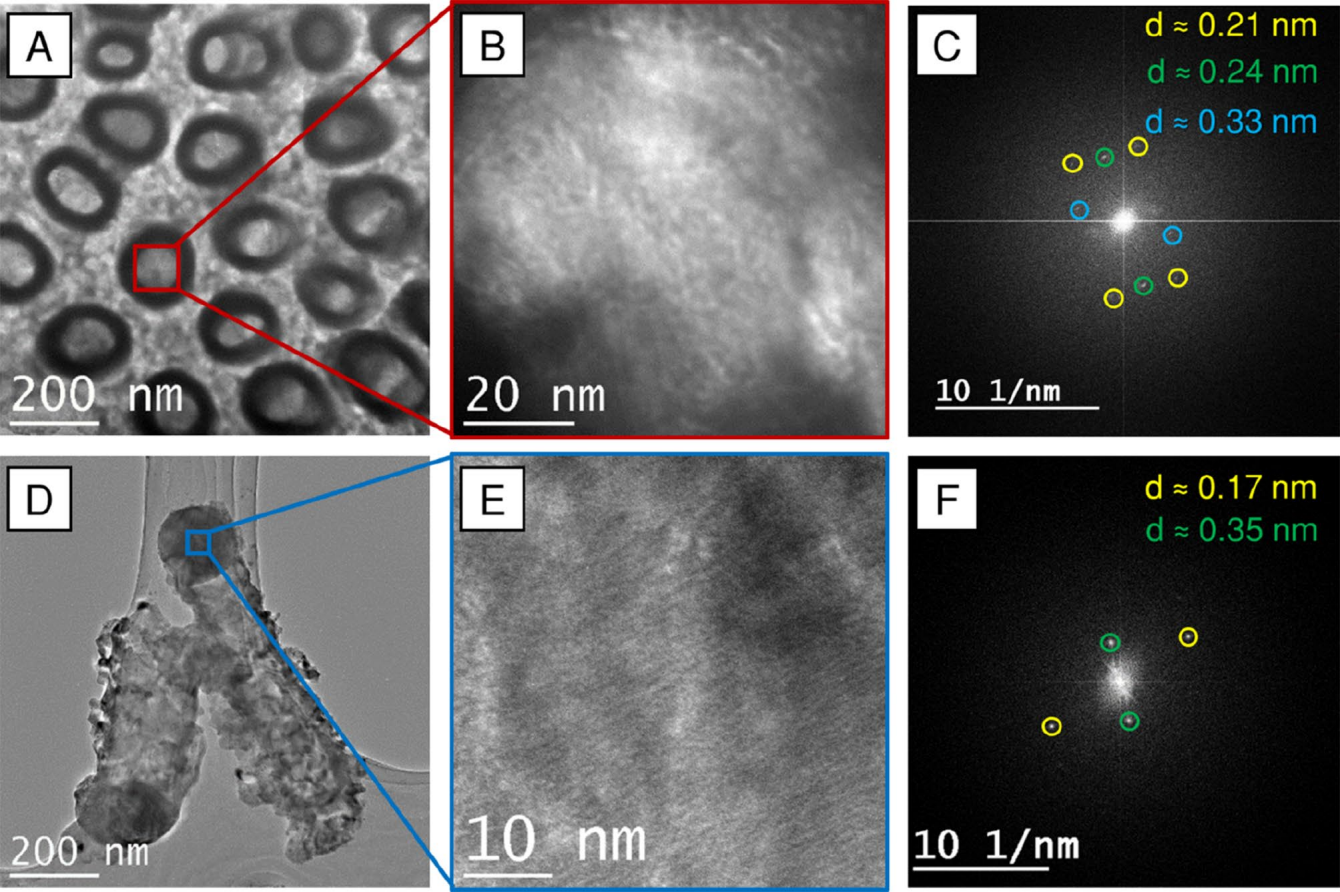
TEM images of the nanotubes irradiated with 30 mJ/cm^2^ with the λ = 355 nm. (**A**) shows the top view of the TiO_2_NTs. (**B**) Close-up of the internal section of the TiO_2_NTs. (**C**) The FFT image of (**B**). (**D**) Shows a removed TiO_2_NT, laying down on the Cu grid allowing for visualization of the cap. (**E**) Shows a close up of the cap and (**F**) the FFT image of Panel E).

The TEM images confirm the empty core of the nanotubes (Fig. 3A) and show a tight cap in their upper region. Notice, that the beam has enough energy to cross the cap and reveal the internal section of the tubes. The images indicate, that the melted cap is approximately 170 nm thick, and there is still nearly 700 nm of the empty nanotube core underneath (Fig. 3D). Moreover, the evidence of the anatase crystalline phase have been found, as the Fast-Fourier Transform of the close-ups of the cap (Fig. 3B,E) reveal *d*_(101)_ = 0.35, *d*_(103)_ = 0.24, and *d*_(105)_ = 0.17 nm peaks (Fig. 3C,F). This shows that the majority of the anatase phase is undisturbed by laser modification.

**Figure 4:**
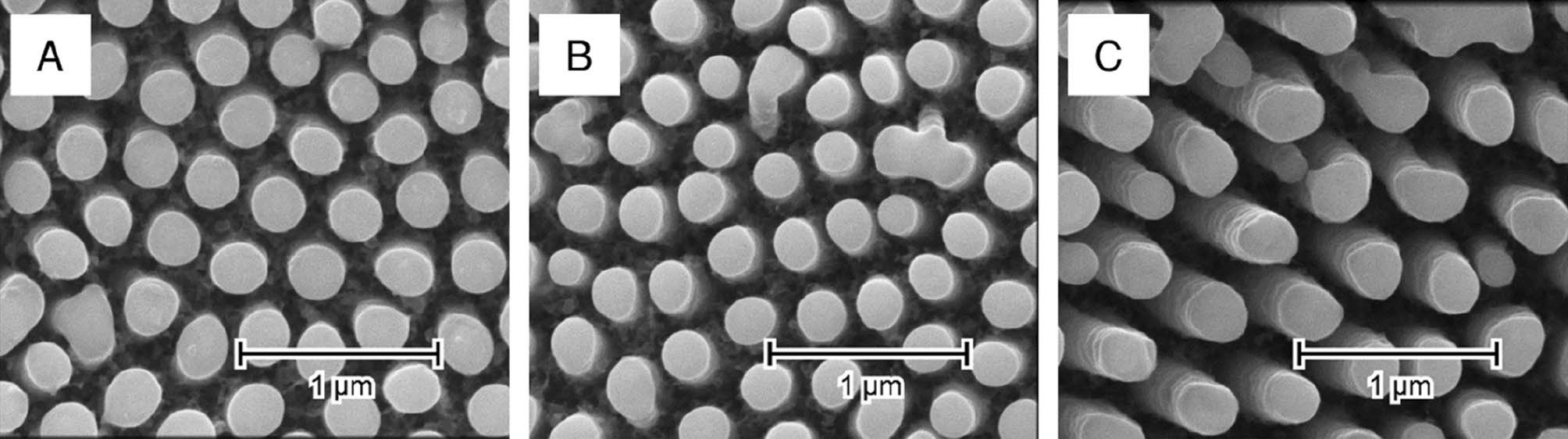
SEM images of the TiO_2_NTs anodized at 40 (**A**), 50 (**B**), and 60 V (**C**) post laser-treatment (λ = 355 nm, 30 mJ/cm^2^).

To our knowledge, such structures have not been yet reported in the literature. Although few works regarding laser-modification of TiO_2_NTs are available^22,23,31,32^, all of the investigators have focused on the densely packed nanotube arrays, where melting of neighboring nanotubes caused their individual features to disappear. Irradiation of differently spaced nanotubes^33^ with λ = 355 nm and 30 mJ/cm^2^, however, is a reliable method of TiO_2_NT encapsulation (Fig. 4).

### Optical properties

#### Reflectance spectra

**Figure 5:**
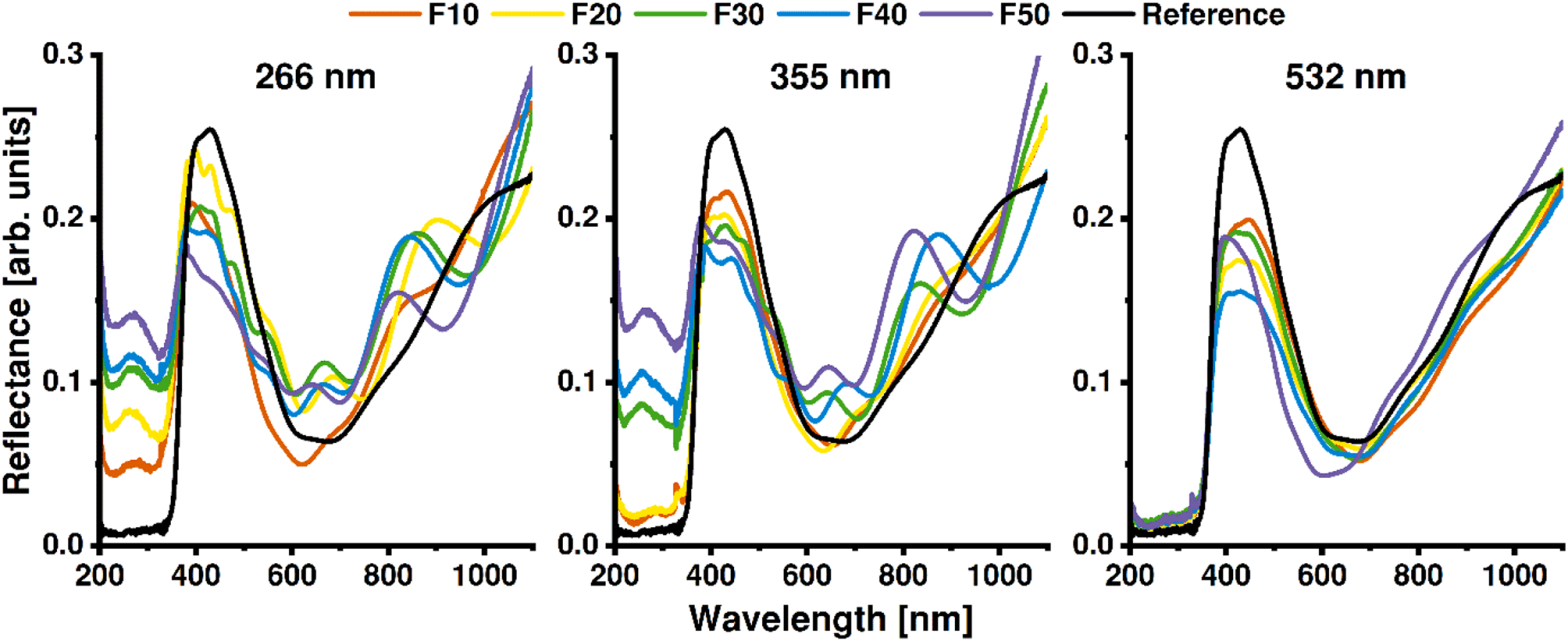
Reflectance spectra of the reference and TiO_2_NTs modified with three different laser wavelengths within the fluence range of 10–50 mJ/cm^2^.

The reflectance spectra for investigated samples are shown in Fig. 5. In the case of modification with λ = 266 and 355 nm, the interference fringes can be seen. They form due to interference of the light-waves reflected from the closed, upper parts of the TiO_2_NTs and those propagating through the TiO_2_NT film, reflected at the bottom. Chiarello et al.^34^ have proposed an equation that allows calculation of the TiO_2_NT length, using two consecutive fringe positions. Regarding the irradiation at a normal angle of incidence, it can be simplified to:

$$d= \frac{{\lambda }_{1}{\lambda }_{2}}{2n({\lambda }_{2}-{\lambda }_{1})}$$

where d is the thickness of the layer, n is the refractive index of the anatase (approximately 2.2 between 450 and 600 nm), and λ_1_ and λ_2_ are the positions of the respective fringes. The equation correlates well into experimental data regarding both geometric and optical features, explaining the periodic nature of the spectrum. Moreover, the presence of the fringes or lack of thereof can be explained by the amount of the material present in the upper nanotube region, i.e. in the cap (Figs. 2, 5). The exception, in this case, would be modification with λ = 355 nm with 10 and 20 mJ/cm^2^, where although capping of the TiO_2_NTs is present, the periodic nature of the spectra is not distinguished. It may be explained by the very thin capping layer and a negligible amount of light reflecting from the cap. Interestingly, whereas Chiarello et al.^34^ observed fringes for spectra recorded for TiO_2_NT layers as thick as 2 µm, we could not define them for the reference material with half the thickness. In this case, the defined spacing between the TiO_2_NTs could be the determining factor responsible for the shape of the reflectance spectra. Interestingly, the changes in the UV region of the reflectance spectra are, except fringes, unrelated to the degree of morphological changes in the TiO_2_NTs (see Fig. 2). An increase in the UV reflectance is present in samples irradiated with 266 and 355 nm lasers. Although in the case of irradiation with 4th harmonics the absorbance drops rapidly, slowing in the upper energy regime, a significant increase in 3rd harmonics is present only when fluence over 30 mJ/cm^2^ is used. As the light absorption in this region is mainly attributed to the electron transitions from O^2−^ orbital to the Ti^4+^ ^35^, an increased presence of oxygen vacancies can result in the rise of radiative recombination seen as the increment in the reflectance spectra.

**Figure 6:**
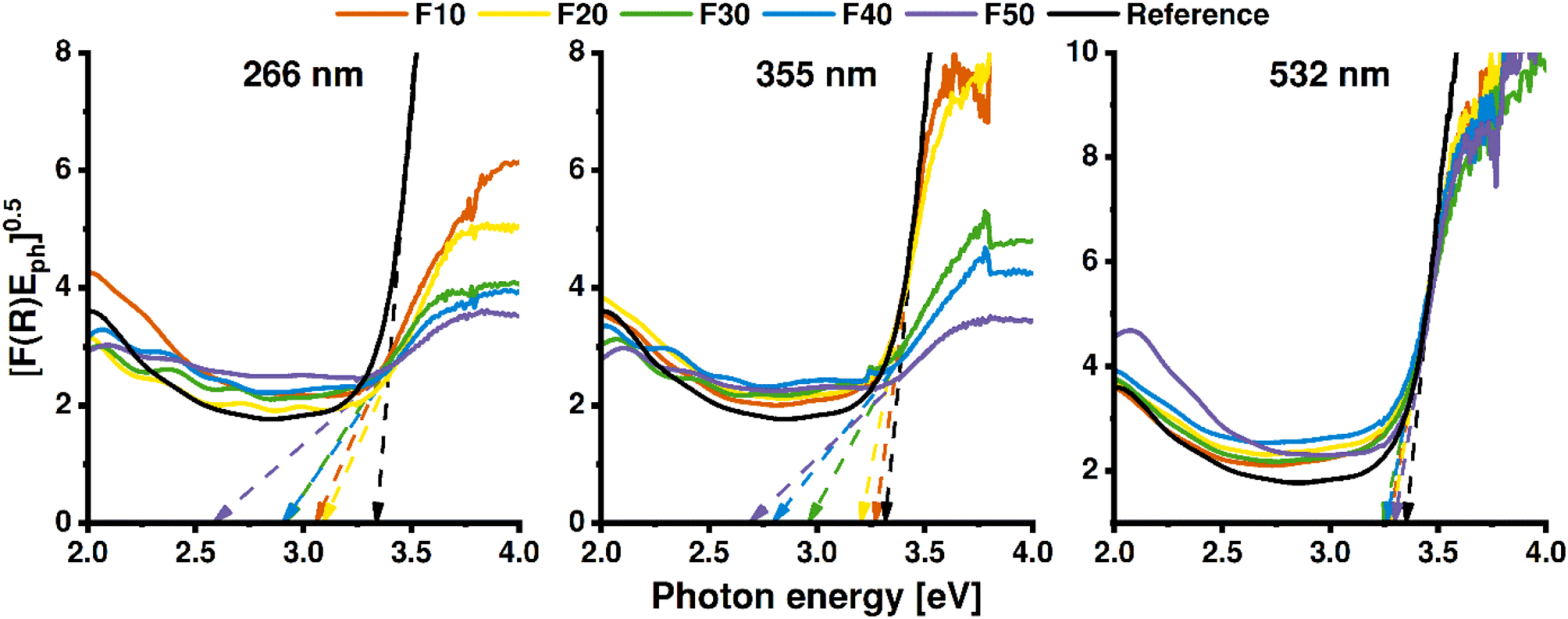
Calculated Tauc plots with dashed lines indicating bandgap values.

Figure 6 shows Tauc plots of the laser-modified TiO_2_NTs accompanied with the untreated sample. The bandgap of the reference sample equals approximately 3.3 eV, slightly higher than for bulk anatase, due to the so-called electron-confinement effect, often present in nanomaterials^36–38^. The E_bg_ shifts towards lower values with increased fluence for both irradiation with 266 and 355 nm, whereas it drops only slightly when the wavelength of 532 nm is applied during the modification. The narrowing of the E_bg_ values is likely related to the creation of additional localized states within the bandgap allowing lower-energy photons to be absorbed^39^.

#### Photoluminescence

**Figure 7:**
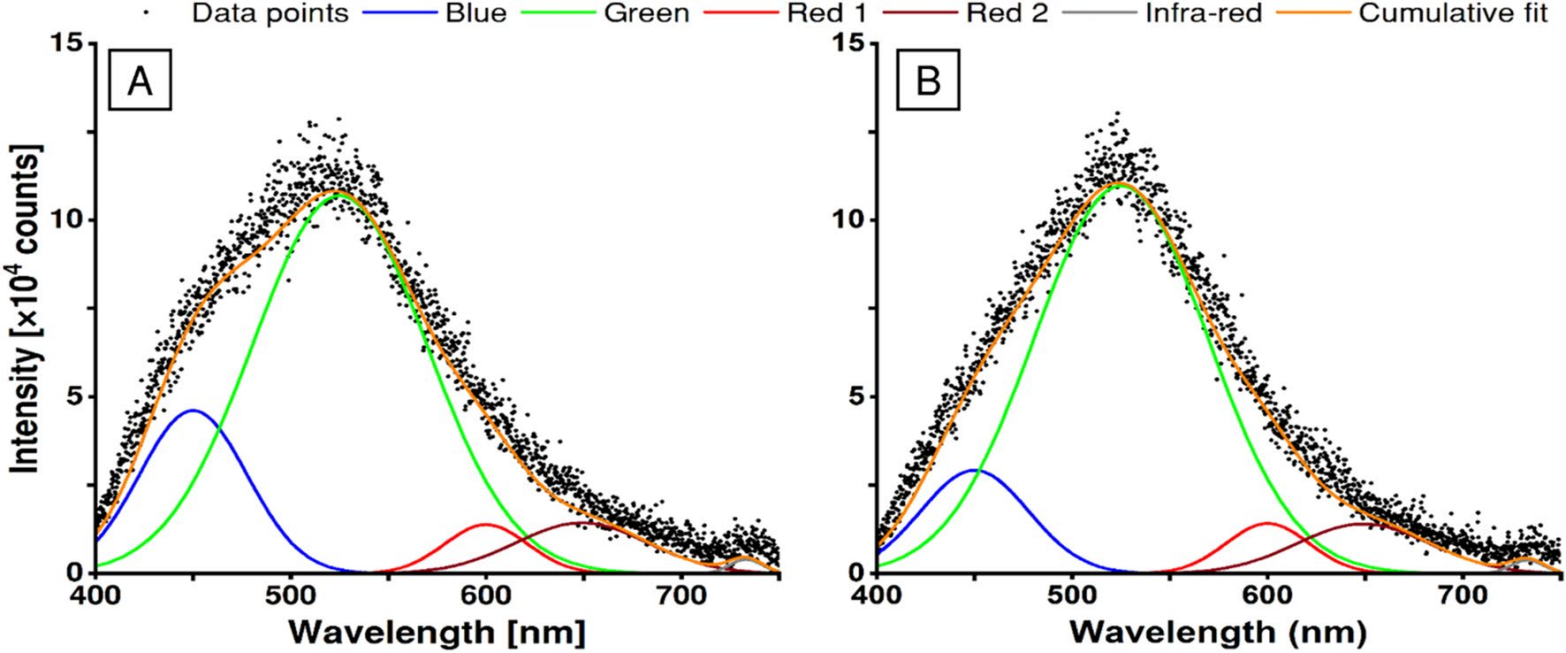
Deconvoluted photoluminescence spectra for unmodified sample (**A**) and the sample modified by 355 nm laser with 30 mJ/cm^2^ (**B**).

Taking into account resolved photoluminescence (PL) spectra reported by other authors, obtained data points were fitted into distinctive emission bands (Fig. 7) using parameters listed in Table 1^40–44^.

**Table 1:** Fitting parameters used to perform deconvolution of photoluminescence spectra.

Colour	Blue	Green	Red 1	Red 2	Infra-red
Peak position [nm]	450	525	600	649	733
FWHM [nm]	65	105	50	80	20

**Figure 8:**
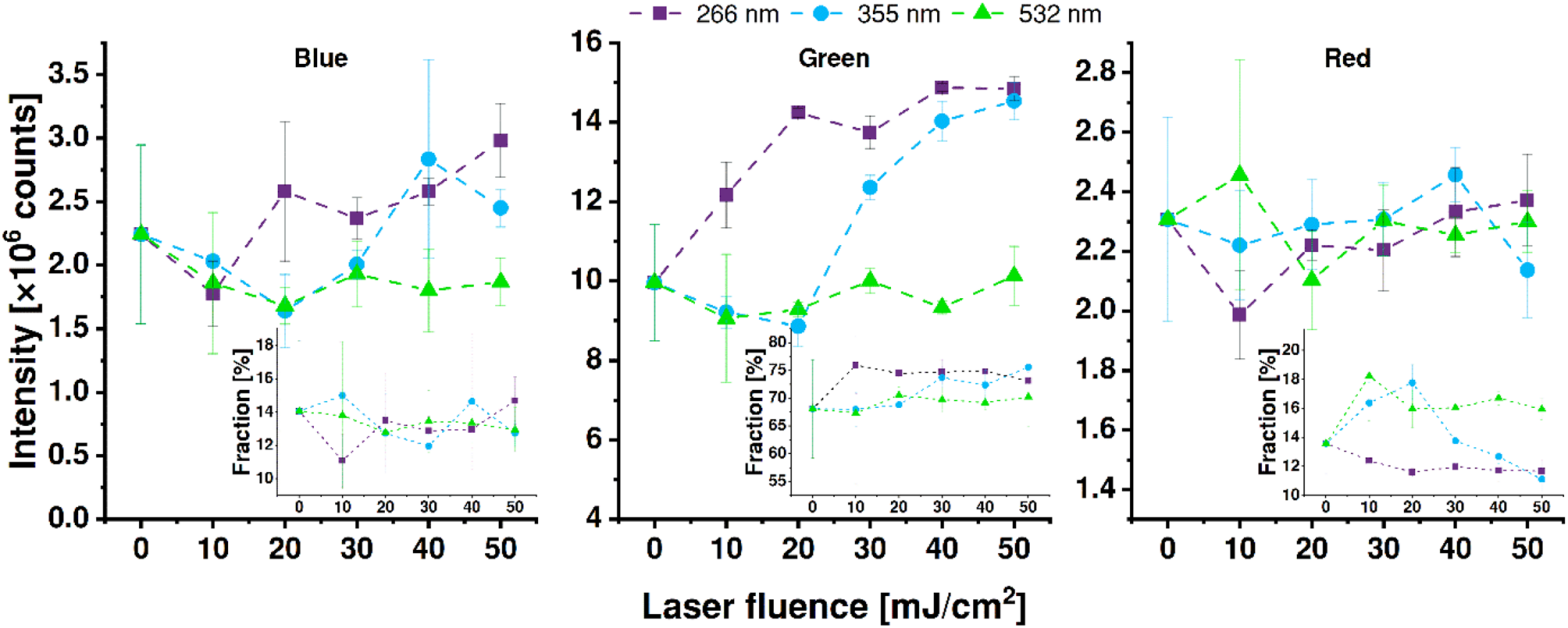
Intensities of the blue, green, and red photoluminescence of the samples as a function of laser fluence used for modification. Insets show the contribution to the total emission.

Analysis of the photoluminescence results, in general, shows that laser annealing of the TiO_2_NTs changes their overall emissivity spectrum. The strongest influence of laser treatment can be observed for the green light emission (Fig. 8) after irradiation of titania with 266 and 355 nm lasers. Although in the case of 4th harmonics the enhancement of emissivity is evident even when the low fluence is used, it quickly saturates at 20 mJ/cm^2^ and a further increase in laser energy does not affect the total green emission. Changes induced by 3rd harmonics are, on the other hand, minimal for fluences up to 20 mJ/cm^2^, but rise and saturate at higher investigated energies. Moreover, a slight increase in the total blue emissivity was observed in the case of modification via both 3rd and 4th harmonics in their respective processing thresholds. However, none of the applied laser wavelengths modulates the red-emission sector and the usage of 2nd harmonics does not noticeably affect the emission spectrum.

In TiO_2_NTs, the blue photoluminescence band is related to electron–hole recombination between conduction and valence bands, green is the effect of recombination of mobile electrons with trapped holes, and red arises due to the recombination of electrons trapped on Ti^3+^ ions with valence band^44,45^. Thus, the increment of the green PL after the treatment with λ = 266 and 355 nm may be explained by the growing number of trapped holes related to the increasing number of oxygen vacancies regarding as the tuning factor of the electronic properties of the TiO_2_ nanostructures^46^. The laser-induced increase of the number of oxygen vacancies was further confirmed by the high-resolution X-ray photoelectron spectra recorded within binding energy of Ti2p (see Figure S1 with appropriate analysis provided in ESI file)^47,48^.

#### Colorimetry

**Figure 9:**
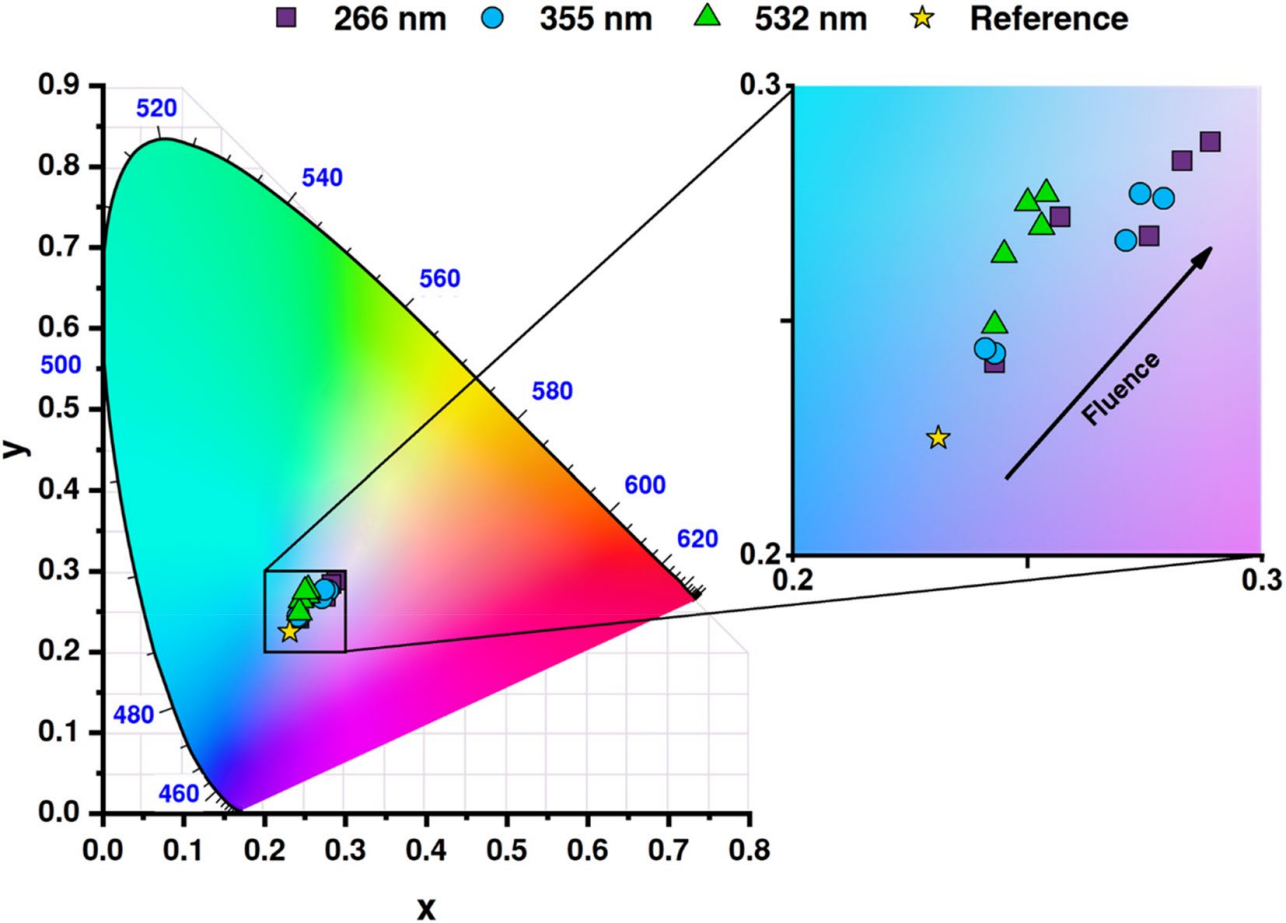
CIE xy chromaticity diagram marked with positions of investigated samples. The inset presents a close-up of the color spectrum and the arrow indicates an increase in fluence.

Apart from the typical analysis of the reflectance spectra, we performed colorimetric analysis and the results were summarized as particular points on a CIE 1931 chromaticity diagram (see Fig. 9). The colorimetric investigation lines up with previously observed effects verified via reflectance measurements. In the series representing the shortest wavelength used for titania treatment, the sample color changes rapidly towards the center of the map (white) and reaching almost the same coordinates at the high-end of measured fluence range. Modification with 355 nm laser pulses affects color only slightly for fluences of 10 and 20 mJ/cm^2^, but the skip on the CIE chart for 30 mJ/cm^2^ is very definitive. Interestingly, the color is changing as well for samples modified with 2nd harmonics. Although no apparent huge shift could be observed via the naked eye, the change from the reference sample towards green color is present and saturates for fluences above 30 mJ/cm^2^. Similar behavior was found for chromium coatings after their exposure to the energetic photons from various laser sources. It confirms that laser modification could be regarded as not fully used, but a powerful tool for optical tuning of thin films^49^. Therefore, without the introduction of any metal or non-metal atoms to the titania structure, optimized laser treatment provides intriguing light-matter interaction behaviors.

## Conclusion

Summarizing, in the frames of this work a unique approach of obtaining free-standing, nanotube-based, hollow titania nanopillars has been shown. In the first step, self-standing titania nanotubes were fabricated via an optimized anodization process, followed by calcination which ensured crystallization. Next, three different wavelengths of a pulsed laser, namely 266, 355 and 532 nm, were used to tailor the upper part of the TiO_2_NTs. Analysis of the SEM/TEM images confirms that the treatment with 355 nm led to the formation of crystalline caps over each hollow pillar, while the photon energies at 266 and 532 nm were either too high or too low resulting in overmelting of the nanotubes or simply preservation of their original geometry. Optical measurements reveal that materials exposed to laser radiation exhibit the nature of photonic crystal. Moreover, the increased number of oxygen vacancies with increased fluence for 266 and 355 nm wavelengths was confirmed by the photoluminescence spectra fitting approach, while colorimetric analysis indicates the possibility of color switching depending on the applied processing parameters. Obtained results strongly suggest that laser interaction with TiO_2_ nanotubes can lead not only to change of their geometrical features but also opens up the possibility of tuning their optical behavior with preserving the crystalline phase and the hollow interior. To the best of our knowledge, such an approach has been shown for the very first time. Additionally, due to the chemical stability of the titania, we believe that prepared material can be adapted for many purposes, especially as a platform for encapsulation of other materials.

## Experimental section

### Sample preparation

Titanium foil (99.7% pure, 0.127 mm thick, Strem) was cut into 35 × 25 mm pieces and degreased ultrasonically for 10 min in acetone (p.a. Protolab), ethanol (96%, Chempur) and deionized (DI) water (0.08 µS, Hydrolab) respectively. After drying in air, it was used as a working electrode in the electrochemical oxidation process to create free-standing TiO_2_NTs, whereas platinum net (25 × 25 mm) was used as a counter electrode. The electrolyte in which both electrodes were submerged consisted of NH_4_F (0.3 wt%, p.a. Chempur), HF (0.5 wt%, p.a. Chempur), and of deionized water (7.0 wt%) in diethylene glycol (p.a. Chempur). Anodization was performed at 30 V, with ramp-up and ramp-down voltages set to 0.1 V/s and controlled by in-house built hardware which was supplied by a constant voltage generator (MPS-600-5L-2). The process was performed at a constant temperature of 40 °C, controlled by a thermostat (Julabo F-12) for 2 h. After anodization, the foil was rinsed and submerged in ethanol for ca. 1 h and dried in air subsequently.

To obtain a crystalline anatase phase, the nanotubes underwent thermal treatment in the furnace (Nabertherm) at 450 °C for 2 h. While the heating rate was set to 2 °C/min, the cooling down to room temperature was performed freely overnight. A sample prepared in such a way was treated as a reference for laser-modified material.

Nd:YAG pulsed laser (6 ns, Quantel) equipped with 2nd, 3rd, and 4th harmonics generator crystals (λ = 266, 355, and 532 nm) was used to modify the surface of TiO_2_NTs structure. The laser repetition rate was set to 2 Hz and its fluence to 10–50 mJ/cm^2^. The beam was homogenized and focused on the surface, creating 2.4 × 2.4 mm spot. The sample was placed on the motorized, computer-controlled stage (SmarAct) enabling precise movement and thus allowing modification on any part of the surface.

### Characterization

Scanning Electron Microscope (SEM) images were taken with FE-SEM, FEI Quanta FEG 250 which was equipped with a secondary electron detector. The pictures were taken at the surface and at the cross-section in multiple places to confirm sample uniformity. During the measurements, a constant acceleration voltage of 10 kV was applied.

Transmission Electron Microscopy (TEM) investigation was done using JEOL ARM 200F, working at 80 kV. Data collected was used to confirm the crystallinity and detailed morphology of the formed nanopillars.

The UV–Vis study was done in the reflectance mode using the PerkinElmer dual-beam spectrophotometer in the range of 300–1100 nm with a scanning speed set to 120 nm/min.

Photoluminescence (PL) spectra were taken using SHAMROCK–SR-303I-A Spectrograph (Andor Technology) at room temperature. It was equipped with 450 mW LED as the excitation light source (λ = 365 nm) and ICCD camera as a detector. FGUV-11 (Thorlabs) and GG 400 (Shott AG) optical filters were used to eliminate unwanted light modes during measurements of the PL spectra. A total of 200 scans in the range of 370–800 nm were done and their average was used for further investigation. The X-ray photoelectron spectroscopy (XPS) studies were carried out using Axis Supra spectrometer equipped with monochromatic AlKα source with 250 µm spot diameter, and 20 eV pass energy, calibrated for adventitious carbon C1s (284.5 eV).

The colorimetric investigation was done using the Konica Minolta CS-2000 spectroradiometer, which allows for the determination of colorimetric coordinates with accuracy and repeatability of ± 0.002 according to CIE xy standard. A halogen bulb (3100 K) was used as a light source, illuminating the sample at 45°, while the measurements were taken perpendicular to the surface from a measuring field of a 1.75 mm in diameter. To become independent of the source spectral characteristics, the Konica Minolta SC-A5 white standard was used for the determination of colorimetric coordinates.

## Supplementary information

Supplementary Information
