# Supplementary material for: Formation of the hollow nanopillar arrays through the laser-induced transformation of TiO2 nanotubes
Source: Sci Rep. 2020 Nov 19;10:20235. doi: 10.1038/s41598-020-77309-2 (PMC7677399; doi:10.1038/s41598-020-77309-2)
Supplement: Supplementary file 1 — Supplementary Information [file 41598_2020_77309_MOESM1_ESM.pdf]

Supplementary file

Formation of the hollow nanopillar arrays through the laser-induced transformation of TiO_2 nanotubes

Jakub Wawrzyniak[1], Jakub Karczewski[2], Piotr Kupracz[1], Katarzyna Grochowska[1], Emerson Coy[3], Adam Mazikowski[4], Jacek Ryl[5], and Katarzyna Siuzdak[1]*

[1] Centre of Plasma and Laser Engineering, Institute of Fluid-Flow Machinery, Polish Academy of Sciences. Fiszera 14 st., 80-231 Gdańsk, Poland

[2] Department of Solid-State Physics, Gdańsk University of Technology, Gabriela Narutowicza 11/12 st., 80-233 Gdańsk, Poland

[3] NanoBioMedical Centre, Adam Mickiewicz University, Wszechnicy Piastowkiej 3 st., 61-614 Poznań, Poland

[4] Department of Metrology and Optoelectronics, Gdańsk University of Technology, Gabriela Narutowicza 11/12 st., 80-233 Gdańsk, Poland

[5] Department of Electrochemistry, Corrosion and Materials Engineering, Gdańsk University of Technology, Gabriela Narutowicza 11/12 st., 80-233 Gdańsk, Poland

*Corresponding author:
jwawrzyniak@imp.gda.pl
Fiszera 14 st.
80-231 Gdańsk
Poland

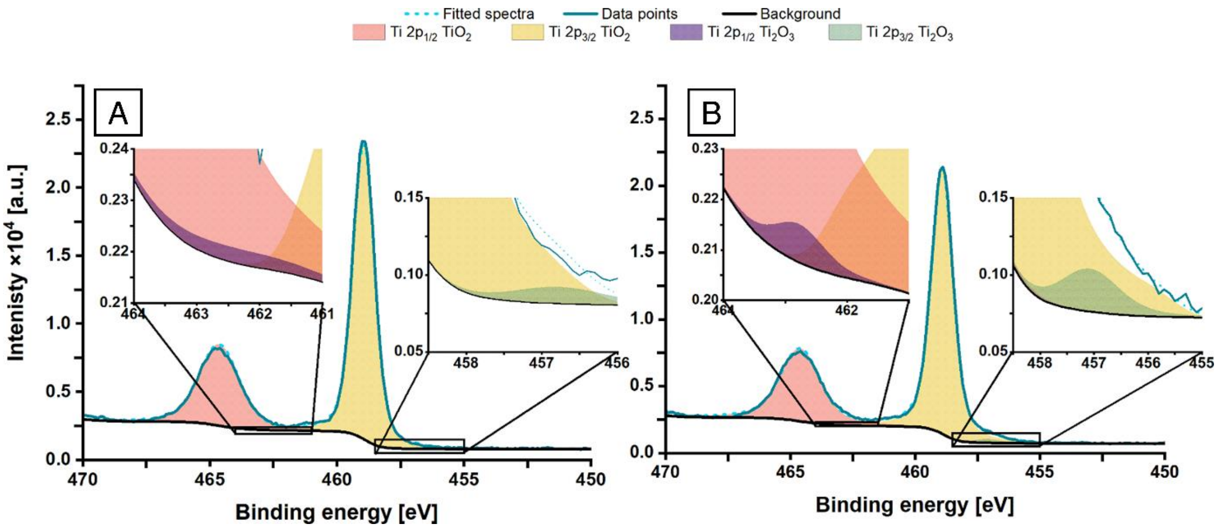

Figure S1: The deconvoluted high-resolution XPS spectra of titanium for (A) bare and (B) laser modified titania nanotubes.

To confirm laser-induced defect generation, the high-resolution XPS spectra were recorded in the binding energy range of titanium. The deconvoluted Ti2p spectra (shown in Figure S1) for the reference sample, and the sample modified with 355 nm laser at 30 mJ/cm^2 consist of two doublets. The primary Ti2p$_{3/2}$ spectral component which can be found at 458.9 eV is characteristic for TiO_2, whereas smaller peak located at 457.1 eV comes from Ti_2O_3 species. The analysis confirms, that the nanotubes are formed primarily of TiO_2, with a small (0.7%) Ti_2O_3 content. The irradiation, however, more than doubles the content of Ti_2O_3 to 1.8%, indicating laser-induced defect generation (*Appl. Surf. Sci.* **357**, 942–950 (2015), *Sci. Rep.* **9**, 12563 (2019)).
